# Structural and mutational studies suggest key residues to determine whether stomatin SPFH domains form dimers or trimers

**DOI:** 10.1016/j.bbrep.2022.101384

**Published:** 2022-11-11

**Authors:** Tomoya Komatsu, Ikuo Matsui, Hideshi Yokoyama

**Affiliations:** a Faculty of Pharmaceutical Sciences, Tokyo University of Science, 2641 Yamazaki, Noda, Chiba, 278-8510, Japan; b Biomedical Research Institute, National Institute of Advanced Industrial Science and Technology (AIST), 1-1-1 Higashi, Tsukuba, Ibaraki, 305-8566, Japan

**Keywords:** Stomatin, SPFH, Oligomerization, *Pyrococcus horikoshii*, Crystal structure, PhStom, stomatin from *Pyrococcus horikoshii*

## Abstract

Stomatin is a major integral membrane protein in human erythrocytes. In a form of hemolytic anemia known as hereditary stomatocytosis, stomatin is deficient in the erythrocyte membrane due to mis-trafficking. It is a member of stomatin, prohibitin, flotillin, and HflK/C (SPFH) domain proteins, and SPFH proteins could function as membrane-bound oligomeric scaffolding proteins in lipid rafts. The previously determined structure of the SPFH domain of *Pyrococcus horikoshii* (Ph) stomatin formed a trimer, whereas that of mouse stomatin formed a dimer. To elucidate the difference of oligomerization state, structural and chromatographic analyses using Ph stomatin were performed, and the key residues were suggested to determine whether SPFH domains form dimers or trimers. From gel-filtration analyses, PhStom (56–234) formed a trimer or tetramer, whereas PhStom (63–234) and PhStom (56–234) K59S formed a dimer. The residues 56–62, particularly Lys59, were involved in trimerization. Based on the crystal structure of PhStom (63–234), it formed a banana-shaped dimer, as observed in mouse stomatin. Thus, residues 162–168 are involved in dimerization. This study provides important insight into the molecular function and oligomerization state of stomatin.

## Highlights

• This study indicated stomatin key residues involved in forming dimers or trimers.
• Oligomerization states were estimated with gel-filtration and the crystal structure.
• Residues 56–62 of PhStom form an archaea-specific trimerization region.
• Residues 162–168 of PhStom form a universal dimerization region.

## 1 Introduction

Stomatin is an integral membrane protein that was first identified in human erythrocytes [1], and is ubiquitously expressed in all tissues [2]. In a form of hemolytic anemia known as hereditary stomatocytosis, the stomatin protein is deficient in the erythrocyte membrane due to mis-trafficking [3]. Stomatin has been shown to modulate the activity of acid-sensing ion channels [4] and GLUT-1 glucose transporter [5,6]. Human stomatin has also been reported to be a major component of vesicles produced by red cells [7], and to potentiate cell fusion [8]. Stomatin is organized into high-order homo-oligomeric complexes of approximately 300 kDa, comprising 9- to 12-mers [9], and is localized in detergent-resistant membrane domains, which are also termed lipid rafts [10,11]. Stomatin is a member of stomatin, prohibitin, flotillin, and HflK/C (SPFH) domain proteins, which are found in the lipid rafts of various cellular membranes [[12], [13], [14]]. SPFH proteins could function as membrane-bound oligomeric scaffolding proteins in lipid rafts [10]. One form of SPFH proteins, prohibitin, has been targeted by small molecules to induce anticancer, cardioprotective, anti-inflammatory, antiviral, and antiosteoporotic activities. Then, modulators of human or bacterial SPFH proteins can be developed to treat a wide variety of human disorders [15].

To date, several structural studies of SPFH proteins have been reported. Our group first determined the crystal structure of the core domain of prokaryotic stomatin PH1511 from the hyperthermophilic archaeon *Pyrococcus horikoshii* (Ph). In the structure, the SPFH domain forms a stable trimer, and three C-terminal α-helical domains extend from the apexes of the triangle [16]. In the solution structure of the SPFH domain of another stomatin, *Pyrococcus horikoshii* PH0470, the SPFH domain formed various oligomers and a multimer even without the coiled-coil region at the C-terminal end [17]. In the first crystal structure of the mouse stomatin as eukaryotes, the SPFH domain assembles into a banana-shaped dimer [18]. As there have been no reports on structures of human stomatin, our group has reported molecular modelling and simulation studies, indicating that human stomatin can oligomerize and approach lipid membranes [19]. Recently, the solution structure of the SPFH domain of human stomatin was determined, and fibril-like self-assembly of the SPFH domain was observed under electron microscopy (EM) [20]. Mutational studies of human stomatin have suggested that the coiled-coil domain was clearly essential for oligomerization, and the cholesterol recognition/interaction amino acid consensus domain was also involved in oligomerization [21]. These reports indicate that the molecular function of the SPFH domain are involved in protein oligomerization as a component of the membrane skeleton [20]. A recent cryo-EM study of HflK/C in complex with FtsH indicated that SPFH domains of HflK and HflC form a ring-like 24 heteromer [22].

SPFH domains tend to form higher-order oligomers. Mouse stomatin forms a banana-shaped dimer, and Ph stomatin forms a trimer as a building block. It remains unclear why mouse and Ph form different building blocks, and which residues are involved in forming a dimer or trimer. Here, we suggest the key residues to determine whether SPFH domains form a dimer or trimer based on structural and chromatographic analyses using Ph stomatin.

## 2 Materials and methods

### 2.1 Construction of expression plasmids

To produce PhStom (56–234) (residues 56–234 of PH1511), the expression plasmid pET-21b (+) (Novagen) containing PhStom (56–234) was used as described previously [16]. To prepare the expression plasmid pET-21b (+) containing a K59S mutant of PhStom (56–234), the polymerase chain reaction (PCR) was carried out using pET-21*b* (+)/PhStom (56–234) as a template. Forward and reverse primers used in the amplification procedure were 5′-ATCTTCGAATCTGCCGTTATCGTAGATTTGAGAACTCA-3′ and 5′-AACGGCAGATTCGAAGATCATATGTATATCTCCTTC-3′, respectively, containing a mutation site (underlined). Using the amplified fragment, the Seamless Ligation Cloning Extract (SLiCE) reaction [23] was performed at 37 °C for 20 min using a similar protocol to that described previously [24]. The SLiCE method is a seamless DNA cloning tool that utilizes homologous recombination activities in *Escherichia coli* lysates to assemble DNA fragments with approximately 15–19-bp homology lengths into a plasmid. Ultracompetent XL2-Blue MRF′ cells (Stratagene) were transformed with the SLiCE solution. The resultant plasmid was purified from the transformant cells.

To prepare the expression plasmid pET-21b (+)/PhStom (63–234), PCR was carried out using pET-21b (+)/PhStom (1–266) as a template [25]. Forward and reverse primers were 5′-GGAATTCCATATGGTAGATTTGAGAACTCAAGTTTTAGACG-3′ and 5′-ATTAATCTCGAGATTGCTCTTATCGCCGGCGACA-3′, respectively, containing *Nde* I and *Xho* I restriction sites (underlined). The amplified fragment was digested with *Nde* I and *Xho* I and ligated into the plasmid pET-21b (+)/PhStom (1–266) between the *Nde* I and *Xho* I sites. Ultracompetent *E. coli* XL2-Blue MRF′ cells were transformed with the ligated product. The resultant plasmid was purified from the transformant cells. The correctness of all plasmids was confirmed by DNA sequencing (Eurofins Genomics). The resultant proteins that can be expressed from these plasmids additionally contain the initial methionine at their N-termini and LEHHHHHH at their C-termini.

### 2.2 Protein expression and purification

*Escherichia coli* BL21 (DE3) Codon-Plus RIL (Stratagene) was transformed with the resultant plasmids. The transformed cells were cultured in 2× yeast tryptone medium containing ampicillin at 37 °C, and induced with 0.5 mM isopropyl β-d-thiogalactopyranoside (IPTG) at 30 °C overnight. The induced cells were centrifuged and stored at −80 °C. Frozen cells were resuspended in a buffer containing 0.1 M Tris-HCl (pH 8.2), 0.3 M NaCl, 5 mM imidazole, protease inhibitor cocktail (Roche), and deoxyribonuclease I (Sigma), and disrupted by sonication on ice. After centrifugation at 27000×*g* for 15 min to remove cell debris, the supernatant was heated at 85 °C for 10 min and centrifuged at 39000×*g* for 15 min to remove denatured proteins.

The supernatant was loaded on Ni-NTA agarose resin (Qiagen) equilibrated with 0.1 M Tris-HCl (pH 8.2) containing 0.3 M NaCl and 5 mM imidazole. After washing with the buffer containing 30 mM imidazole, proteins were eluted with a buffer containing 0.1 M NaCl and 500 mM imidazole. The eluate was applied to a Hi Load 16/60 Superdex 200 gel-filtration column (GE Healthcare) equilibrated with a buffer containing 50 mM Tris-HCl (pH 8.2) and 50 mM NaCl with a flow rate of 1 mL/min using an AKTAprime plus system (GE Healthcare). An approximate molecular mass of the elution peak was estimated by the elution time of ovalbumin (44 kDa), conalbumin (75 kDa), aldolase (158 kDa), ferritin (440 kDa), and thyroglobulin (669 kDa) of the gel filtration calibration kit (GE Healthcare). The resultant protein was concentrated using an Amicon ® Ultra-15 centrifugal concentrator (Merck). Purification was done within two days.

### 2.3 Crystallization

Crystallization was performed with the sitting-drop vapor-diffusion method at 20 °C. Crystallization drops were prepared manually by mixing 0.5 μL protein solution and 0.5 μL reservoir solution. The purified PhStom (63–234) was 10 mg/mL in 50 mM Tris-HCl (pH 8.2) and 50 mM NaCl. The reservoir solution contained 0.1 M sodium acetate, 50 mM Tris-HCl (pH 8.5), and 15% PEG4,000. Needle-like imperfect crystals appeared, and were improved by microseeding to generate rod- or needle-shaped crystals suitable for X-ray data collection with an approximate size of 0.5 × 0.03 × 0.03 mm.

### 2.4 Data collection and structure determination

A crystal was cryoprotected in a solution of 35% (v/v) glycerol in the reservoir solution, and flash-cooled in a nitrogen-gas stream at 95 K. X-ray diffraction data were collected on beamline BL-17A of the Photon Factory in KEK (Tsukuba, Japan) with an Eiger X16 M detector (Dectris), and then integrated and scaled with *XDS* [26] and *SCALA* [27]. The structure was determined by the molecular replacement method with the program *MOLREP* [28] in the *CCP4* suite [29] using the structure of residues 63–170 of chain A of PhStom (56–234) (PDB ID: 3BK6) as a search model [16]. The model was subjected to several cycles of crystallographic refinement with *REFMAC5* [30], followed by manual model building and fitting with *COOT* [31]. Data collection and refinement statistics are summarized in Table 1. Interface areas were calculated with *PDBePISA* (*Proteins, Interfaces, Structures and Assemblies*) from the European Bioinformatics Institute (https://www.ebi.ac.uk/msd-srv/prot_int/pistart.html) [33]. Least-squares fitting of two structures was performed with *LSQKAB* in the *CCP4* suite or *PDBeFold* (https://www.ebi.ac.uk/msd-srv/ssm/ssmstart.html) [34]. All molecular figures were produced with *PyMOL* (http://www.pymol.org/). The atomic coordinates and structure factors have been deposited in the Protein Data Bank Japan (PDBj) with the accession code 8GN9.

**Table 1:** Data collection and refinement statistics.

Data collection	
PDB ID	8GN9
Wavelength (Å)	0.98000
Space group	*P*4_1_2_1_2
Cell dimensions	
*a*, *b*, *c* (Å)	28.01, 28.01, 240.63
Resolution range (Å)	20.00–2.50 (2.64–2.50)^a^
No. of observed reflections	40,442
No. of unique reflections	3909 (536)
*R*_merge_ (*I*)^b^	0.140 (0.311)
Completeness	0.997 (1.000)
CC_1/2_^c^	0.995 (0.951)
Average *I*/σ	10.6 (6.8)
Wilson *B*-factor (Å^2^)	31.3

*Refinement*	

Resolution range (Å)	19.81–2.50
Completeness	0.994
No. of reflections used	3443
*R*^d^/*R*_free_^e^	0.215/0.278
No. of non-hydrogen atoms	
Protein	840
Solvent	22
Average *B* factors (Å^2^)	
Protein	45.5
Solvent	38.7
R.m.s. deviations from ideality	
Bond lengths (Å)	0.007
Bond angles (°)	1.381
Ramachandran plot^f^ (%)	
Favored region	93.3
Allowed region	6.7
Outlier region	0

a Values in parentheses are for the highest-resolution shell.

b *R*_merge_ (*I*) = Σ_*hkl*_Σ_*j*_ | *I*_*j*_ (*hkl*) − <*I* (*hkl*)> |/Σ_*hkl*_Σ_*j*_*I*_*j*_ (*hkl*), where *I*_*j*_ (*hkl*) is the intensity of an individual reflection and <*I* (*hkl*)> is the mean intensity of that reflection.

c CC_1/2_ is Pearson's correlation coefficient between the measured intensities of two randomly assigned half-subsets of reflections in the overall dataset.

d *R* = Σ_*hkl*_ | |*F*_obs_| − |*F*_calc_| |/Σ_*hkl*_ |*F*_obs_|, where |*F*_obs_| and |*F*_calc_| are the observed and calculated structure factor amplitudes, respectively.

e *R*_free_ is calculated for 10% of the reflections randomly excluded from refinement.

f Values were calculated with *MolProbity* [32].

## 3 Results and discussion

### 3.1 Putative trimeric region of SPFH domains

**Fig. 1:**
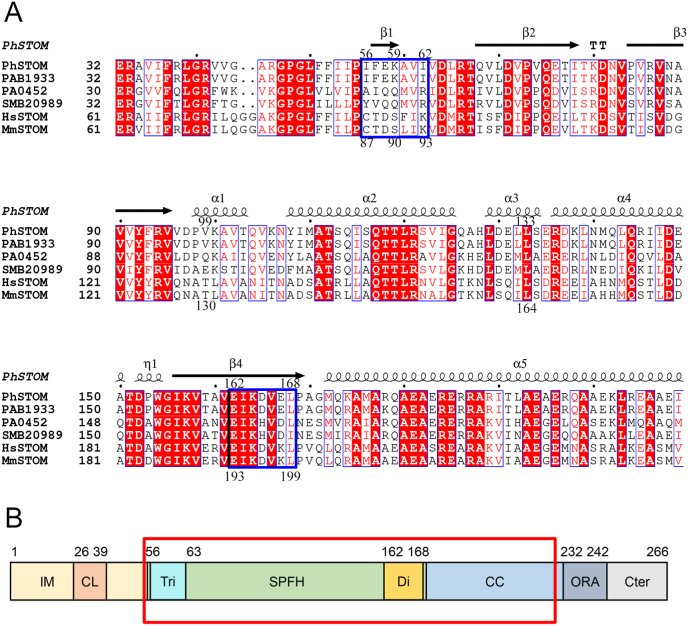
Sequence alignment and domain arrangement of stomatin. (A) Sequence alignment of the SPFH domain of stomatin. The sequences were aligned with the program *ClustalW* (https://www.genome.jp/tools-bin/clustalw) and displayed along with the secondary-structure assignment for the PhStom (56–234) structure (PDB ID: 3BK6) with the program *ESPript* (https://espript.ibcp.fr/ESPript/ESPript/) [35]. α, α-helix; β, β-strand; η, 3_10_-helix; TT, β-turn. A black dot above the sequence marks every 10 amino acids. White letters boxed with a red background indicate residues that are conserved in these sequences, and red letters boxed with a thin square indicate similar residues. Protein names and UniProt entries are as follows: PhSTOM (*P. horikoshi* stomatin PH1511, O59180), PAB1933 (*P. abyssi* stomatin, Q9V0Y1), PA0452 (*Pseudomonas aeruginosa* stomatin, Q9I666), SMB20989 (*Rhizobium meliloti* stomatin, Q92UL3), HsSTOM (human stomatin, P27105), MmSTOM (mouse stomatin, P54116). Blue bold boxes show residues involved in tri- or dimerization, and numbers at the top and bottom of the sequence are residues of PhSTOM and MmSTOM, respectively. (B) Domain arrangements of PhSTOM. Abbreviations are as follows according to a previous report [21]: IM, intramembrane domain; CL, cholesterol recognition/interaction amino acid consensus (CRAC)-like motif; CC, coiled-coil domain; ORA, oligomerization and lipid raft-association domain; Cter, C-terminal domain. Tri or Di indicate the region involved in tri- or dimerization in the SPFH domain. We previously determined the structure of the region (residues 56–224) shown as a red box in PhStom (56–234) [16]. (For interpretation of the references to color in this figure legend, the reader is referred to the Web version of this article.)

As indicated, SPFH domains of PhStom form a trimer [16], whereas those of mouse stomatin form a dimer [18]. To understand the reason for the difference, we focused on the residues involved in the inter-subunit interactions of PhStom (56–234) (Fig. S1A). Residues 56–62 and 162–169 are involved in inter-subunit interactions. Among them, the side chain of Lys59 shows electrostatic interactions with the side chains of Asp165 and Glu167 (Fig. S1B). Sequences of SPFH domains of stomatin of archaea, bacteria, and eukaryotes were aligned (Fig. 1A). Residues 56–62 of PhStom (residues 87–93 in mouse) are not conserved, whereas residues 162–168 (residues 193–199 in mouse) are well-conserved. In mouse stomatin, residues 193–202 are involved in the formation of a banana-shaped dimer [18]. However, residues 87–93 are not needed to form the dimer. Then, Fig. 1B shows the expected domain arrangements of PhStom. Residues 56–62 of PhStom correspond to a trimerization region that is not essential, and residues 162–168 correspond to a dimerization (or trimerization) region that is essential. Trimerization by the residues 56–62 may be specific for archaea.

### 3.2 Gel-filtration analyses indicate residues 56–62 are involved in trimerization

**Fig. 2:**
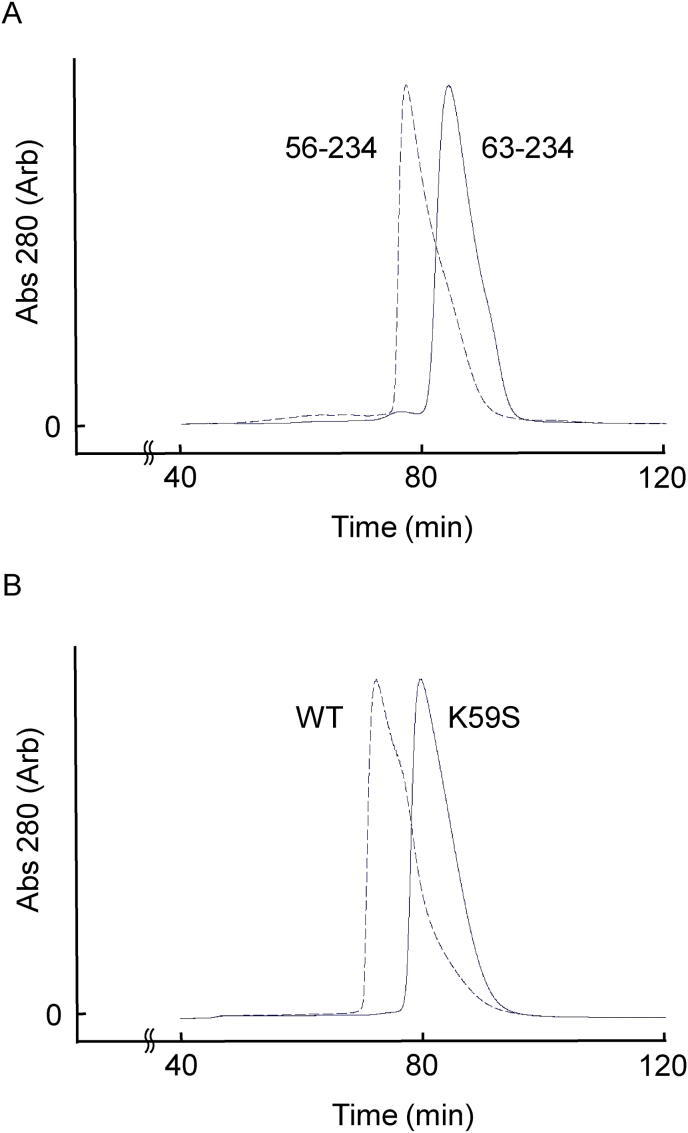
Elution profile of PhStom on a Superdex 200 gel filtration column. (A) Difference of PhStom (56–234) shown as a dashed line and PhStom (63–234) shown as a solid line. (B) Difference of WT (dashed line) and K59S mutant (solid line) of PhStom (56–234).

To verify the hypothesis that residues 56–62 of PhStom are involved in trimerization, gel-filtration analyses were performed using PhStom (56–234), PhStom (63–234), and a K59S mutant of PhStom (56–234) (Fig. 2). From the elution profile (Fig. 2A), the estimated molecular mass of PhStom (56–234) was 79 kDa, indicating that it forms a trimer or tetramer based on the molecular mass of its monomer, 21 kDa. On the other hand, the estimated molecular mass of PhStom (63–234) was 44 kDa, indicating that it forms a dimer based on the molecular mass of its monomer, 20 kDa. Then, Lys59 of *P. horikoshii* is aligned to Ser90 of humans and mice (Fig. 1A). Thus, PhStom (56–234) K59S is a mutant of eukaryotic-type stomatin. The estimated molecular mass of PhStom (56–234) K59S was 46 kDa, and thus it forms a dimer (Fig. 2B). These results indicate that loss of residues 56–62 or introduction of K59S mutation prevents trimerization and instead causes dimerization.

### 3.3 Structure determination of PhStom (63–234)

To verify the oligemerization state of the PhStom (63–234) or PhStom (56–234) K59S mutant, crystallization trials were performed. Using a crystal of PhStom (63–234), the structure was determined by the molecular replacement method. Since the structure of PhStom (56–234) (PDB ID: 3BK6) contains residues 56–224 in chain A, we first tried molecular replacement using a search model of residues 63–224 of chain A of 3BK6. However, the molecular replacement was unsuccessful. If the asymmetric unit contains one PhStom (63–234) molecule of 21 kDa, the Matthews coefficient *V*_M_ value is 1.2 Å^3^ Da^−1^, which is too small. If the molecule is truncated to the residues 63–170 of 12 kDa, the *V*_M_ value is 1.9 Å^3^ Da^−1^, which is within standard *V*_M_ ranges of protein crystals [36]. Then, we tried molecular replacement using a search model of residues 63–170, which is devoid of the coiled-coil domain, and could determine the structure (Table 1).

The structure of PhStom (63–234) contains one molecule in an asymmetric unit. The final model contains residues 64–169, one Na^+^, and 21 water molecules. There are no vacant spaces in the crystal to occupy the remaining residues 170–234. Therefore, it is considered that the coiled-coil region of residues 170–234 was degraded during crystallization. It is also considered that the coiled-coil region is flexible, as discussed in the previous report [16], and is susceptible to degradation by proteases.

### 3.4 PhStom (63–234) forms a banana-shaped dimer

**Fig. 3:**
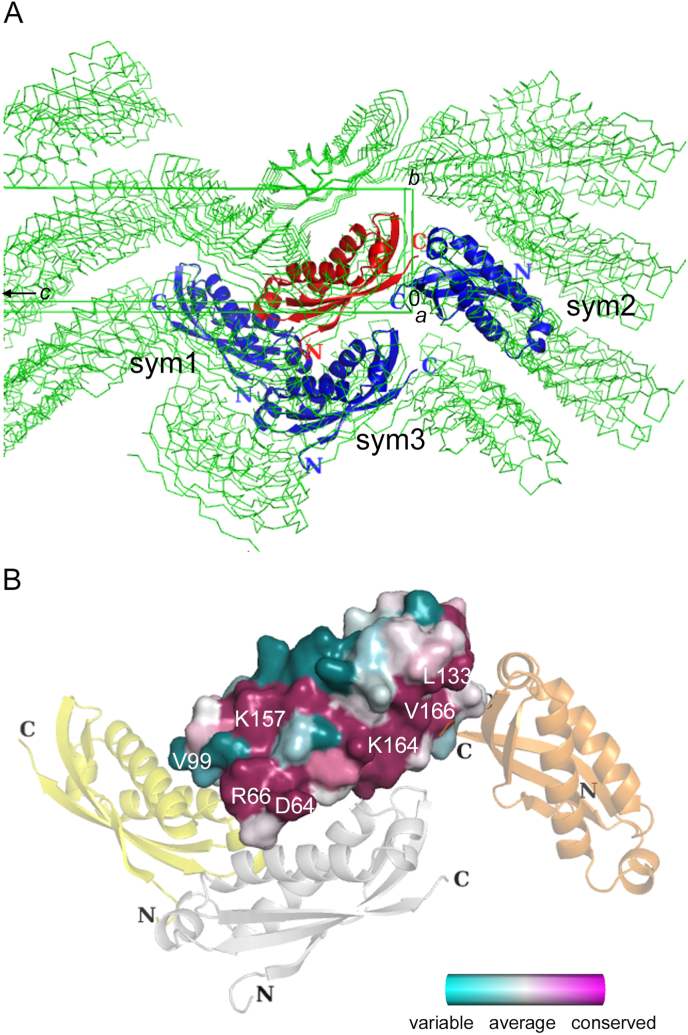
The structure of PhStom (63–234). (A) Crystal packing of PhStom (63–234). The structure of the original monomer is colored red, and other symmetry-related molecules from the original monomer are colored green. Based on the original monomer, neighboring symmetry-related monomers with a large interface area are shown as blue and labeled sym1, sym2, and sym3, according to the results of *PDBePISA*. A unit cell box is also shown. N and C denote N- and C-termini of each monomer, respectively. (B) Surface representation of PhStom (63–234). Sequence conservation with variable (cyan) and conserved (magenta) residues is mapped onto the surface. The figure was produced with *Consurf* (https://consurf.tau.ac.il/) using the aligned sequences of Fig. 1A [37]. The view is similar to that in Fig. 3A. Symmetry-related monomers of sym1, sym2, and sym3 are colored yellow, orange, and grey, respectively. (For interpretation of the references to color in this figure legend, the reader is referred to the Web version of this article.)

The structure of PhStom (63–234) consists of an SPFH domain (residues 64–169) and is nearly the same as that of the SPFH domain of PhStom (56–234) [16]. The root-mean-square deviation (rmsd) value for all Cα atoms is 1.2 Å. Although the value is relatively large, the rmsd of residues 68–168 is 0.6 Å. To analyze the oligomerization state, the interface areas between PhStom (63–234) and its symmetry-related molecules were calculated. As shown in Fig. 3A, the molecule was closely arranged in a thin and long crystal lattice (*a* = *b* = 28 Å, *c* = 241 Å). Neighboring symmetry-related molecules with the largest interface area are shown in descending order as sym1 (N-terminal head to N-terminal head interaction), sym2 (C-terminal tail to C-terminal tail interaction), and sym3 (side to side interaction), with areas of 494, 418, and 335 Å^2^, respectively (Table S1). Sequence conservation was calculated using stomatin of six species of archaea, bacteria, and eukaryotes (Fig. 3B). Low-conserved residues such as Val99 are located in the interface of sym1 (Table S1), and thus the interaction may be not universal to stomatin species. Although conserved residues such as Asp64 and Arg66 are located in the interface of sym3, its interface area is small. Conserved residues such as Leu133, Lys164, and Val166 are located in the interface of sym2. This C-terminal tail to C-terminal tail interaction causes the formation of the banana-shaped dimer as reported in mouse stomatin [18]. The structure of the banana-shaped dimer (original monomer and sym2) is similar to that of mouse stomatin (PDB ID: 4FVF) and the rmsd of aligned 203 residues is 1.5 Å (Fig. S2). Relatively-conserved residues 162–168 are also important for forming a banana-shaped dimer in archaea in addition to eukaryotes.

## 4 Conclusions

This study investigated the key residues involved in oligomerization of the SPFH domain of stomatin. Our previously determined structure of PhStom (56–234) shows a trimer, and the candidate residues involved in trimerization are residues 57–62, mainly Lys59. Gel-filtration analyses using PhStom (56–234), PhStom (56–234) K59S, and PhStom (63–234) were performed. PhStom (63–234) forms a dimer, whereas PhStom (56–234) forms a trimer or tetramer. PhStom (56–234) K59S forms a dimer. From these results, residues 56–62 contribute to forming a trimer, and Lys59 is particularly involved in trimerization. The crystal structure of PhStom (63–234) contains residues 64–169, and it forms a banana-shaped dimer in the crystal packing. Residues 162–168 are involved in dimerization. This banana-shaped dimer has been observed in mouse stomatin [18]. From these results, residues 162–168 are universally involved in dimerization (or trimerization), and residues 56–62 are archaea-specifically involved in trimerization. This study might contribute to elucidating the oligomerization state of the SPFH domain.

### Author statement

HY designed the research. TK performed experiments. TK and HY determined structures. IM and HY analyzed the data. TK and HY wrote the manuscript.

## Funding sources

This work was supported by 10.13039/501100001691JSPS KAKENHI Grant Number 17K07316 to H.Y. from the 10.13039/501100001700Ministry of Education, Culture, Sports, Science and Technology (10.13039/501100001700MEXT).

## Declaration of competing interest

The authors declare no conflicts of interest.

## Data Availability

The data that has been used is confidential.
