# Supplementary material for: Structural and mutational studies suggest key residues to determine whether stomatin SPFH domains form dimers or trimers
Source: Biochem Biophys Rep. 2022 Nov 11;32:101384. doi: 10.1016/j.bbrep.2022.101384 (PMC9663324; doi:10.1016/j.bbrep.2022.101384)
Supplement: Multimedia component 1 [file mmc1.docx]

**Supporting Information**

**Structural and mutational studies suggest key residues to determine whether stomatin SPFH domains form dimers or trimers**

Tomoya Komatsu,^a^ Ikuo Matsui,^b^ and Hideshi Yokoyama^a,^*

^a^ Faculty of Pharmaceutical Sciences, Tokyo University of Science, 2641 Yamazaki, Noda, Chiba 278-8510, Japan

^b^ Biomedical Research Institute, National Institute of Advanced Industrial Science and Technology (AIST), 1-1-1 Higashi, Tsukuba, Ibaraki 305-8566, Japan

*Corresponding author: Hideshi Yokoyama

Email: yokoyama@rs.tus.ac.jp; Tel.: +81-4-7121-3663

Table S1. Interface area and interfacing residues of PhStom(63-234)


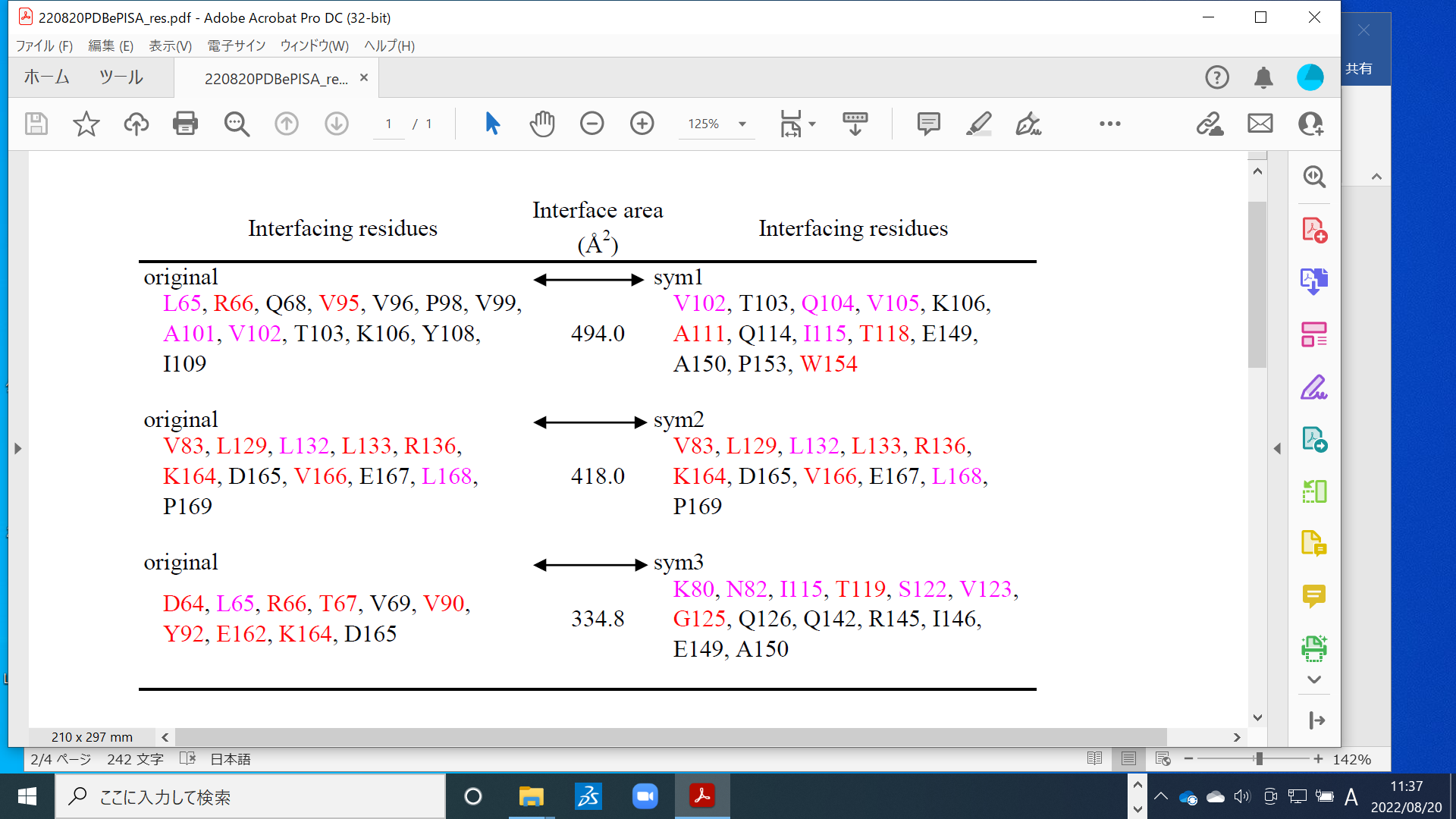


Interface areas between PhStom(63-234) and its symmetry-related molecules were calculated with *PDBePISA*. Among six species of stomatin shown in Fig.1A, perfectly-conserved residues are colored red, relatively-conserved residues are colored magenta, and low-conserved residues are colored black.


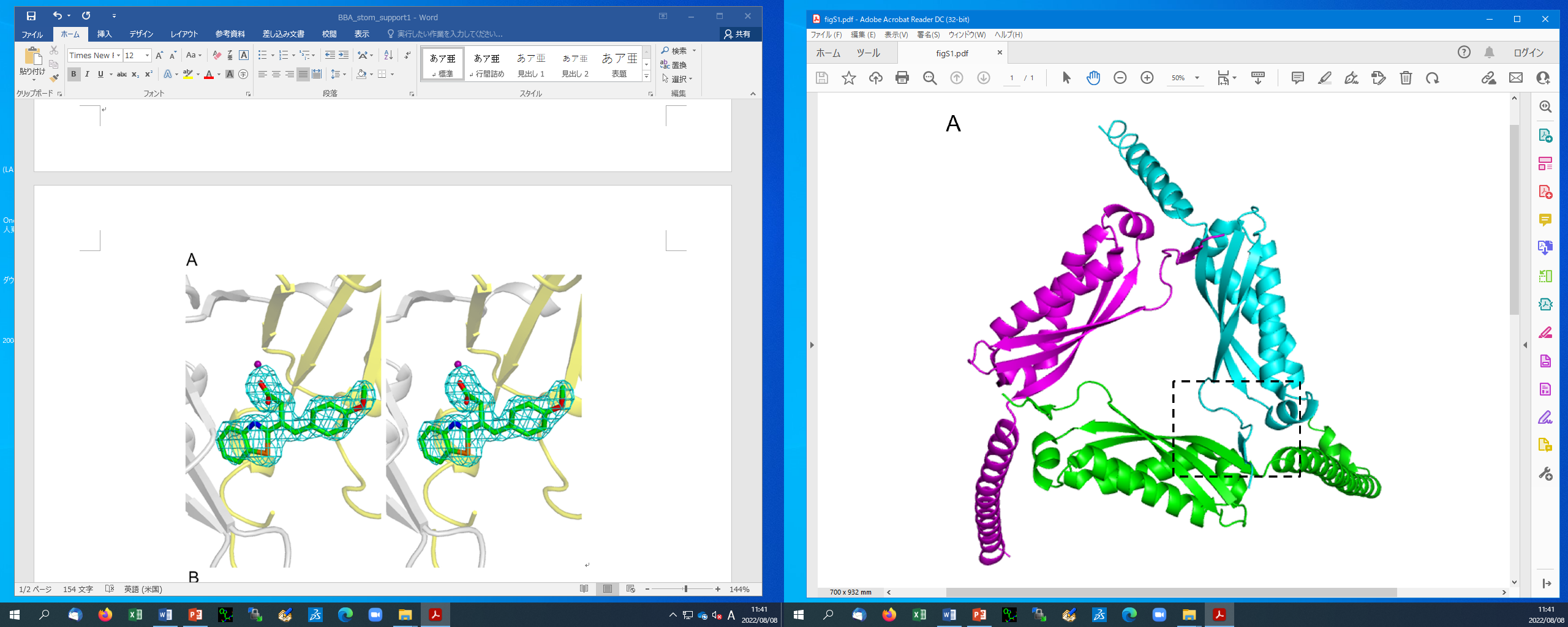


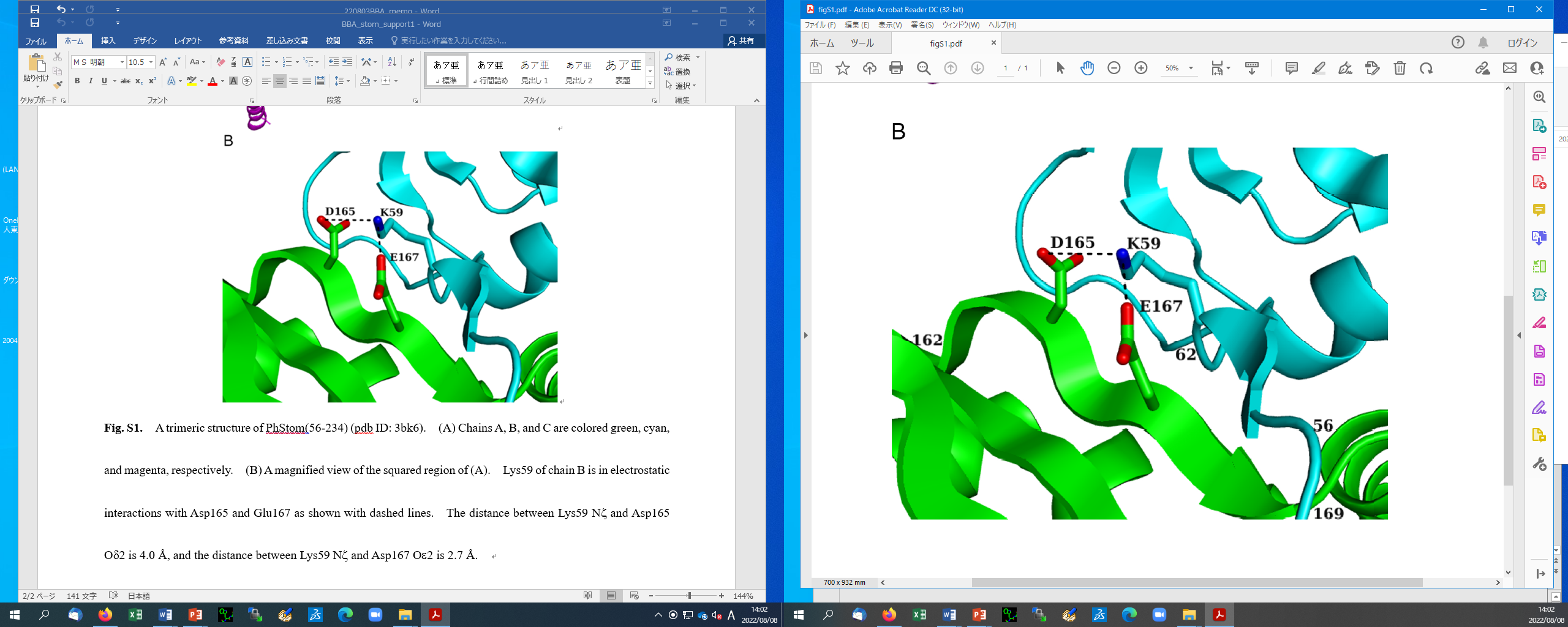


Fig. S1. A trimeric structure of PhStom(56-234) (pdb ID: 3bk6). (A) Chains A, B, and C are colored green, cyan, and magenta, respectively. (B) A magnified view of the squared region of (A). Lys59 of chain B shows electrostatic interactions with Asp165 and Glu167 of chain A, as shown with dashed lines. The distance between Lys59 Nζ and Asp165 Oδ2 is 4.0 Å, and the distance between Lys59 Nζ and Asp167 Oε2 is 2.7 Å. Residues 56-62 and 162-169 are involved in inter-subunit interactions, and the residue numbers of Cα atoms are labeled.


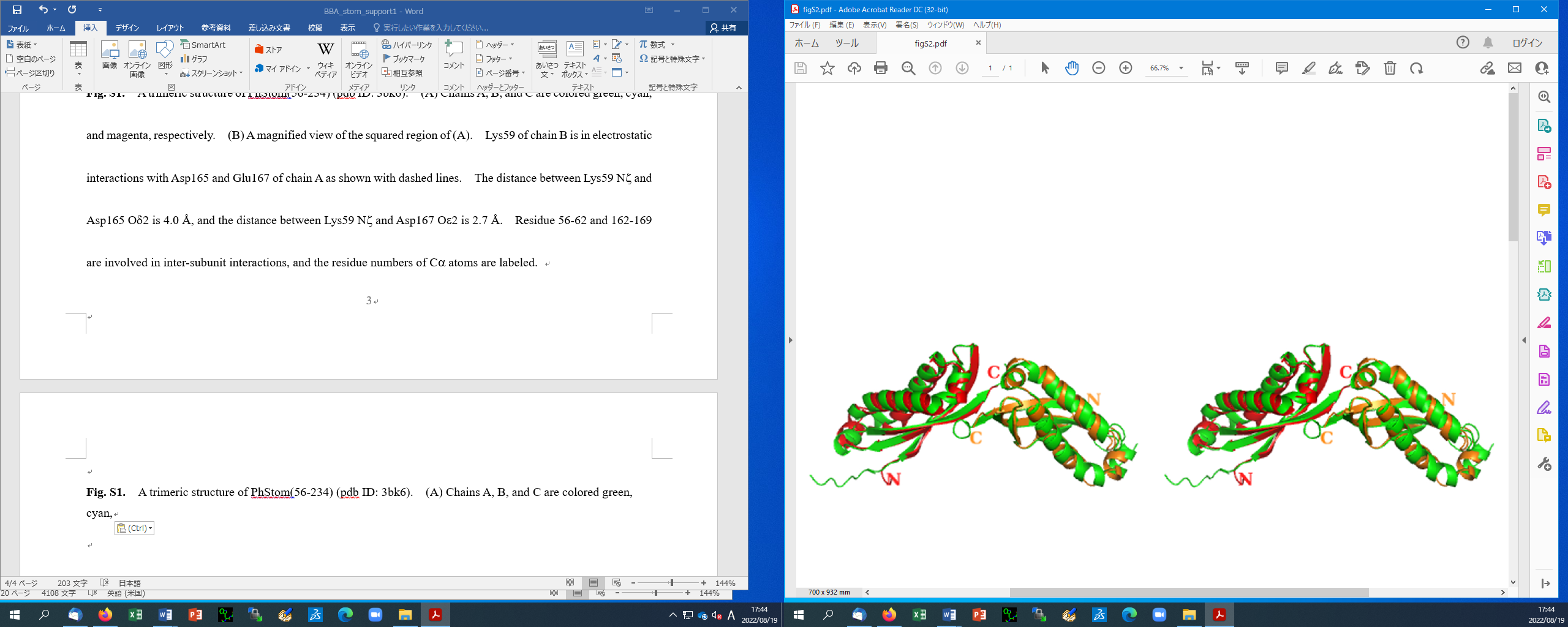


Fig. S2. Stereo representation of the superposed dimer of PhStom(63-234) and mouse stomatin. The original monomer of PhStom(63-234) is colored red, and a symmetry-related molecule (sym2, calculated with *PDBePISA*) of PhStom(63-234) is colored orange. N and C denote N- and C-termini of each monomer, respectively. The dimer of mouse stomatin (PDB ID: 4FVF) is colored green. Two dimers were superposed with *PDBeFold*.
